# Smooth Pursuit Eye Movement of Monkeys Naive to Laboratory Setups With Pictures and Artificial Stimuli

**DOI:** 10.3389/fnsys.2018.00015

**Published:** 2018-04-17

**Authors:** Yehudit Botschko, Merav Yarkoni, Mati Joshua

**Affiliations:** Edmond and Lily Safra Center for Brain Sciences, The Hebrew University of Jerusalem, Jerusalem, Israel

**Keywords:** animal behavior, learning, eye movement, smooth pursuit, motion

## Abstract

When animal behavior is studied in a laboratory environment, the animals are often extensively trained to shape their behavior. A crucial question is whether the behavior observed after training is part of the natural repertoire of the animal or represents an outlier in the animal’s natural capabilities. This can be investigated by assessing the extent to which the target behavior is manifested during the initial stages of training and the time course of learning. We explored this issue by examining smooth pursuit eye movements in monkeys naïve to smooth pursuit tasks. We recorded the eye movements of monkeys from the 1st days of training on a step-ramp paradigm. We used bright spots, monkey pictures and scrambled versions of the pictures as moving targets. We found that during the initial stages of training, the pursuit initiation was largest for the monkey pictures and in some direction conditions close to target velocity. When the pursuit initiation was large, the monkeys mostly continued to track the target with smooth pursuit movements while correcting for displacement errors with small saccades. Two weeks of training increased the pursuit eye velocity in all stimulus conditions, whereas further extensive training enhanced pursuit slightly more. The training decreased the coefficient of variation of the eye velocity. Anisotropies that grade pursuit across directions were observed from the 1st day of training and mostly persisted across training. Thus, smooth pursuit in the step-ramp paradigm appears to be part of the natural repertoire of monkeys’ behavior and training adjusts monkeys’ natural predisposed behavior.

## Introduction

Studying any behavior in the lab creates a conflict between the need to control behavior precisely and the advantages of observing natural behavior. Field studies reveal a great deal about natural tendencies but make it difficult to standardize experiments, manipulate the environment and measure behavior accurately. On the other hand, training animals in the lab on a very specific task with associated specific demands may recruit specialized mechanisms that might not generalize to other environments. The conundrum between natural and restricted environments is especially critical in neuroscience where the goal is to probe the neural basis of behavior. Recording neural activity imposes strong constraints on the experimental environment. For example, in many studies the head is restrained to record neural activity at high temporal and spatial resolution (Evarts, 1968; Fuchs and Luschei, 1970). Thus, natural behavior which for instance involves coordination between head and eye or body is restricted.

The rationale for studying the neural basis of behavior in animals is to be able to observe and deduce principles that generalize within and across species (Joshua and Lisberger, 2015). Ultimately, the goal is to bridge from animals to the human brain. A major concern is that studying the neural mechanisms related to a specialized behavior may preclude generalizing between species or even between animals. Thus, one of the key issues is whether a behavior studied in the lab is part of the natural repertoire of the experimental animal. This can be assessed by evaluating the time it takes for a behavior to be learned and the conditions under which it is expressed early in training.

We used the smooth pursuit eye movement system to study how monkeys learn to track moving targets in a lab setup. The eye movement generally (Robinson, 1981) and the smooth pursuit system specifically (Krauzlis, 2004) have been widely used as a model system in neuroscience. Although humans and monkeys exhibit the same kind of behavior upon presentation of a similar visual stimulus (Fuchs, 1967) there are differences in how we train humans and monkeys. A single verbal description of the task is often sufficient to instruct humans to perform eye movement tasks, whereas monkeys are often extensively trained over months or even years prior to data collection (Shichinohe et al., 2009). The relevance of the animal model thus depends on determining how well monkeys perform these tasks in the initial period of behavioral measurements.

Recent studies have shown that in the initial phase of training, the smooth eye movements are small and that across weeks of training monkeys’ performance improves markedly. Bourrelly et al. (2016) showed that during the early exposure to moving targets, tracking was composed of relatively large saccades separated by very low gain (defined as the eye/target velocity) smooth pursuit episodes. Over several days of training, pursuit velocity increased while the amplitude and frequency of saccades decreased. Hietanen et al. (2017) reported that training markedly enhanced the ocular following response of naïve monkeys across several week of training. Here, to study whether the low gain of pursuit in the initial training days was a result of the specific stimulus condition, we attempted to optimize the pursuit stimuli. First, we used the step-ramp paradigm (Rashbass, 1961) to drive initiation of movement with smooth pursuit. Second, in addition to a small spot that is often used in lab setups for pursuit tasks, we used larger stimuli that drive pursuit more effectively (Heinen et al., 2016; Mukherjee et al., 2017). Finally, we introduced pictures of monkeys that might engage the natural tendency of monkeys to track moving stimuli since they are both naturally relevant to the monkey and more closely resemble the stimuli observed ecologically.

We monitored the monkeys’ behavior during the initial days of training. We found that in some conditions the monkeys could track targets with fast smooth eye movements as early as the 1st day of training. In these conditions the monkeys tracked the target primarily through pursuit accompanied by small corrective saccades. Pictures of monkeys were tracked with the highest eye velocity. The small spot stimulus evoked slow eye movements in the initial days but improved markedly over 12–14 days of training (Bourrelly et al., 2016). Extensive training lasting several months led to a small additional increase in pursuit speed. Thus overall, pursuit movement appeared to be part of the natural repertoire of these monkeys. The large pursuit velocity from day one onward and the rapid improvement throughout training suggest that the basic sensory and motor structures that drive pursuit exist in naïve monkeys.

## Materials and Methods

Data were collected from two male Macaca fascicularis monkeys (4–5 kg, 4–5 years old). All procedures were approved in advance by the *Institutional Animal Care and Use Committees* of the Hebrew University of Jerusalem and were in strict compliance with the *National Institutes of Health Guide for the Care and Use of Laboratory Animals*. Monkeys were first trained to sit calmly in a primate chair (Crist Instruments) and to consume food from a tube in front of them. To prepare the monkeys for recording eye movements, we implanted a headholder on the skull to allow us to restrain the monkeys’ head movements. The location of the eye was measured with a high temporal resolution camera (1 KHz, Eye link – SR research) and collected for further analysis.

Visual stimuli were displayed on a CRT monitor (1280 pixels × 1024 pixels, refresh rate of 85 Hz) at 45–60 cm from the monkeys’ eye. The stimuli appeared on a dark background in a dimly lit room. A computer performed all real-time operations and controlled the sequences of target motion. We used three types of targets: a bright white circle measuring 0.5° in diameter, pictures of monkeys, and a scrambled version of the same monkey pictures. Initially we trained the monkeys on a single picture and verified the results in later sessions using additional pictures. In monkey B we used a 2° × 2° picture and switched between different pictures with the same size on the 13th day. In monkey C we switched from a 2° × 3° to a different 3° × 2° picture on the 6th training day and confirmed the results in a later session with a 2° × 2° picture (not shown). To scramble the pictures, we randomly shuffled the picture pixels. The shuffling procedure preserved the picture size, luminance and colors but removed all spatial correlations between pixels and left no noticeable objects in the picture.

During the initial training the monkeys learned to direct their gaze toward a still target to obtain a liquid food reward (baby food mixed with water and infant formula). In these initial stages the target was presented in different locations on the screen, typically along the horizontal and vertical axis at locations with eccentricities of 0, 5, 10, and 15 degrees. We trained the monkeys for 5–7 days until they fixated for up to 2 s on the target. We used spots and pictures to train the monkeys to associate the fixation with reward. For monkey B we also used the scrambled pictures for training and employed them from Day 1 of the step-ramp paradigm. For monkey C we only used the scrambled pictures during Day 2 of training on the step-ramp paradigm. To calibrate the eye signal we adjusted the horizontal and vertical offset and gain while presenting the stimulus in the center of the screen and at locations 15° eccentric to the horizontal and vertical axis. The window and duration of fixation during calibration varied across days; namely, the window was typically around 5° and the duration was around 1–2 s. We used either pictures or spots for calibration. Using large stimuli for calibration might lead to inaccurate calibration, which could be detected by large differences in the center of fixation for large and small stimuli. However, we found that there was only a small difference between the center of gaze for spots and pictures. Specifically, the average distance between the center of gaze for spots and pictures was 0.6° (±0.17°, range [0.36° 0.89°]) and 0.43° (±0.14°, range [0.2° 0.72°]) for Monkeys C and B. The small differences in center of gaze of the different targets indicated that we could calibrate the equipment with either stimulus, thus ensuring sufficient accuracy for the smooth pursuit experiment.

At the start of each step ramp trial, a stationary target appeared in the middle of the screen and the monkeys were required to fixate within an invisible 4° × 4° window. This large window allowed the monkeys to fixate on features in the picture that were not in the center of the screen. Although the window was large, the monkeys typically fixated on the target and made only small fixational movements. For monkey C the standard deviation of the eye position was 0.3° and 0.4° (horizontal and vertical components) for the spot condition and 0.65° and 0.58° for the picture condition. For monkey B the values were 0.47° and 0.75° for the spot and 0.46° and 0.78° for the picture. Further, the eyes were directed outside a 2° × 2° window around the center of fixation for less than 5% of the time (although this was consistent with 75% of the permitted gaze direction). Prior to motion the target appeared in the center of the screen for 700–1500 ms and then the same target jumped to a location 4° eccentric to the center of the screen (step) and then moved at 20°/s (ramp) toward the center of the screen (Rashbass, 1961). In the first 150 ms after target motion onset, the monkeys had a grace period during which we withheld the accuracy requirements. After this period, the monkeys were required to keep their gaze within a 3–7° invisible square window around the target. Failure led to aborting of the trial. Different stimuli and different motion directions were interleaved in the same block and the order of trials was randomized.

We used large windows to avoid restricting the monkeys’ behavior to accurate pursuit. The large window (>3°) only led to failures after the initiation of pursuit, thus confirming that we could collect data from trials in the period just after target motion onset even when the pursuit was substantially slower than target velocity. The window was limited in size (<7°) to force the monkeys to move their eyes to complete a trial successfully. Including or excluding trials in which the monkeys failed to match the accuracy requirements did not change any of the findings. The target continued to move at a constant speed for 700 ms (except for the first session for monkey C when the motion duration was 900 ms) and then stopped. The monkeys were required to continue to fixate for 400–600 ms and then received a liquid food reward (0.15–0.25 ml).

We used eye velocity and acceleration thresholds to detect saccades automatically and then verified the automatic detection by visual inspection of the traces. The velocity and acceleration signals were obtained by digitally differentiating the position signal after we smoothed it with a Gaussian filter with a standard deviation of 5 ms. Saccades were marked as eye acceleration larger than 1000°/s^2^, eye velocity crossing 15°/s during fixation or eye velocity crossing 50°/s while the target moved. To calculate the average of the smooth pursuit initiation we first removed the saccades and treated them as missing data. We also used the detected saccades to calculate the fraction of pursuit displacement on a trial. In each trial we calculated overall eye displacement as the amplitude of the eye position in the direction of target motion. The pursuit displacement was calculated as the sum of eye position displacements between the times of the saccades. The fraction of pursuit displacement was then calculated as the ratio of the pursuit displacement to the total eye displacement. Using the target displacement (Bourrelly et al., 2016) or the total eye displacement in the denominator did not alter the findings.

We decomposed the eye velocity traces into latency and constant acceleration. We constructed a template of zeros in the first 75 ms which afterward rose linearly with a slope of 1 for 100 ms. We fit this template to the eye velocity by shifting it in time and scaling it. The shift and scale parameters that resulted in the largest *r*-square provided an estimate of the latency and constant acceleration of the eye. We report the values for the fit of the average traces. Fitting the average traces explained more than 99% of the variance in all sessions and all target conditions except for the 1st day of the spot condition for monkey C in which the fit explained 77% of the variance. We opted to run this algorithm on the average traces since parameter estimation from single trial traces could be very noisy especially when the eye movement gain was small. Nevertheless, using single trials instead of averages did not alter the findings.

For image power spectral analysis, we calculated the Fourier transform of the picture and the scrambled stimulus (van der Schaaf and van Hateren, 1996; Torralba and Oliva, 2003), $F\;(u,\;v)\;=\sum_{x,y}^{}I\;(x,\;y)e^{-2\pi i(ux/N+vy/M)}$ where, *u* and *v* are the spatial frequency coordinates in the horizontal and vertical directions, *I* is the image intensity normalized between zero and one and *N* and *M* are the number of horizontal and vertical pixels in the picture. We calculated the power spectrum of each image as: $S\;(u,\;v)\;=\frac{{\left|F(u,v)\right|}^{2}}{L}$ where *L* is the number of pixels in the image. To calculate the average power across spatial frequencies we converted *u* and *v* to polar coordinates (*f*, φ) with *u*=*f* cos φ and *v*=*f* sin φ. The average power was calculated across all frequencies in bins of 1 cycle/image. We present the power spectrum of the grayscale image. We confirmed that for the features we extracted from the pictures there was not any substantial difference between the different pictures we used or between different color components of the same picture.

## Results

### Smooth Pursuit Eye Movements During the Initial Stages of Training on a Step-Ramp Paradigm

**FIGURE 1:**
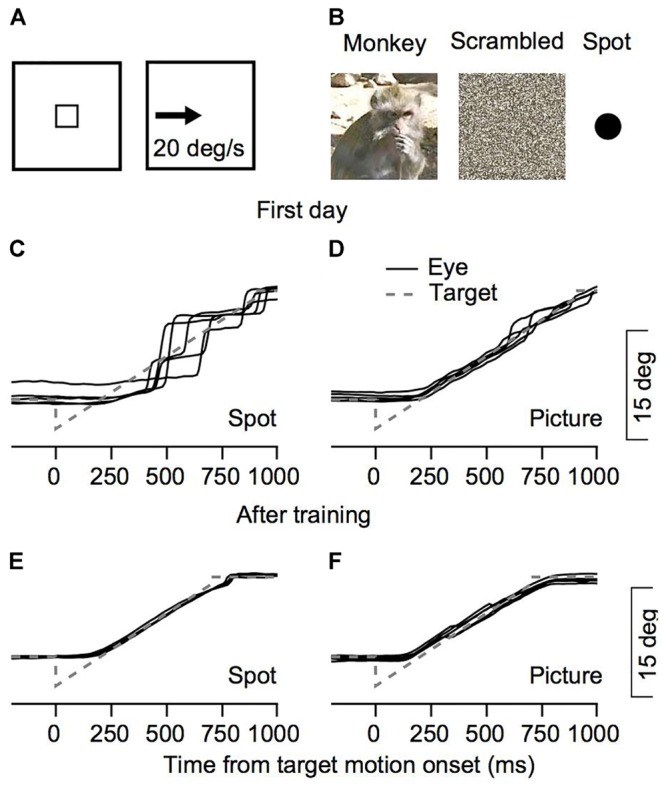
Behavioral task and examples of eye movements. **(A,B)** The snapshots illustrate the temporal structure of the task. The small square rectangle represents a target that was presented in the center of the screen which stepped to an eccentric position after a short delay and moved at constant speed. Three possible targets were interleaved in each session: a picture of a monkey, a scrambled version of this picture, or a spot. **(C–F)** Examples from Monkey C for eye position traces from the 1st day of training **(C,D)** and after extensive training **(E,F)**. Solid black lines represent the eye position in the five trials in which Monkey C tracked a target that moved to the left. To insure these were not outliers we chose five trials where the rank of the fraction of pursuit displacement out of the total eye displacement was closest to the median. The gray dashed lines represent the target position.

We recorded eye movements from the 1st day of training on a step-ramp paradigm (Rashbass, 1961). At the beginning of each trial, the monkeys were required to fixate on a target in the center of the screen (**Figure 1A**). After a short delay the target jumped to a position 4° eccentric to the center of the screen (step) and started to move at 20°/s toward the center of the screen (ramp). We presented the moving stimuli after the monkeys were trained to fixate on targets (5–7 days of training).

To study eye movements during the initial steps of training, we recorded eye movements from the 1st day of training on the monkey picture stimulus, the scrambled version of the picture and the bright spots (**Figure 1B**). **Figures 1C–F** present examples of traces of eye positions from the first training session for monkey C and after extensive training for 6 months. When the tracking target was a spot, movement was mostly composed of large saccades (**Figure 1C**). When the monkey tracked a moving picture, the eye continuously followed the picture and only small saccades were required to correct for errors (**Figure 1D**). After extensive training the monkey followed the target continuously both when tracking the spot and the picture (**Figures 1E,F**). Thus, the examples in **Figure 1** show differences in the tracking of a picture of a monkey and a spot as early as the 1st day of recording.

**FIGURE 2:**
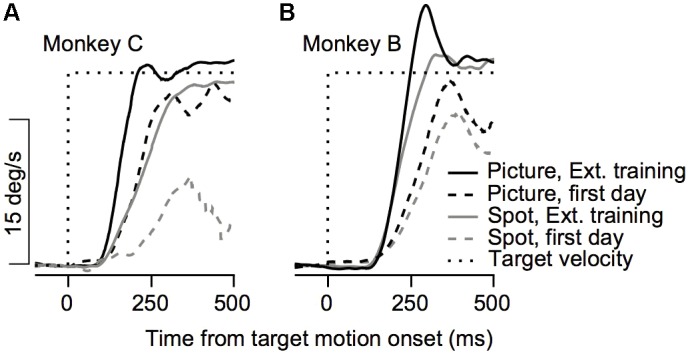
Example of the effect of training on initial eye velocity. Average eye velocity as a function of the time from target motion onset for Monkey C **(A)** and Monkey B **(B)**. The dashed and solid traces show data from the 1st day of training and after extensive training. The black and gray traces show the eye velocity for trials in which a picture and a spot were presented as the target. Dotted black line represents the target velocity. Data are shown for Monkey C and B on trials in which the target moved to the left and to the right, respectively.

**FIGURE 3:**
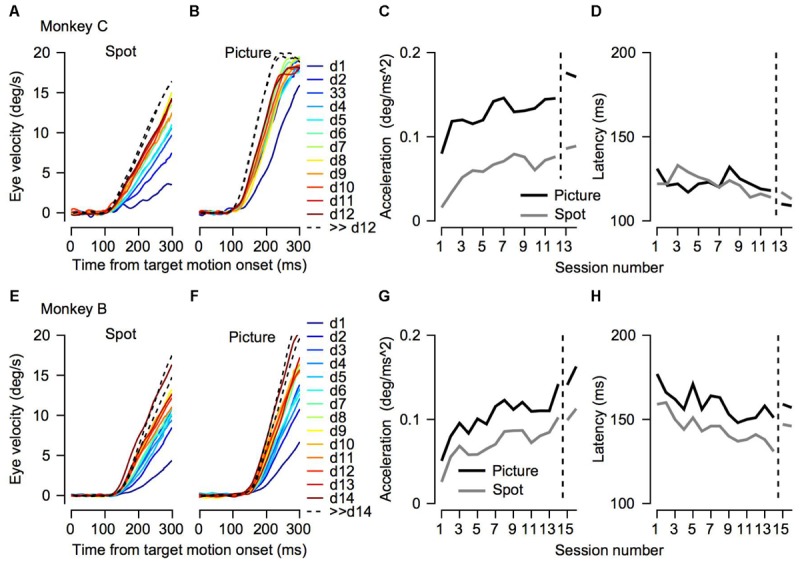
Initial eye velocity traces across training sessions. **(A,B,E,F)** Eye velocity as a function of time from target motion onset. Each thin line shows the average eye velocity in a single session. Colors correspond to the color code on the right and represent the session number. The progression from dark blue through green to dark red corresponds to the session number starting from the 1st day of training. The black dashed traces show the data from sessions that were recorded after many days of training on pursuit tasks. Different columns correspond to the different target conditions. **(C,D,G,H)** Decomposition of the eye velocity traces to the slope of linear regression (linear acceleration; **C,G**) and latency **(D,H)**. The horizontal axis represents the session number. Values to the right of the vertical dotted line were recorded after extensive training on pursuit tasks. Presented values were extracted from the average traces. The top **(A–D)** and bottom **(E–H)** plots shows data for Monkeys C and B.

**FIGURE 4:**
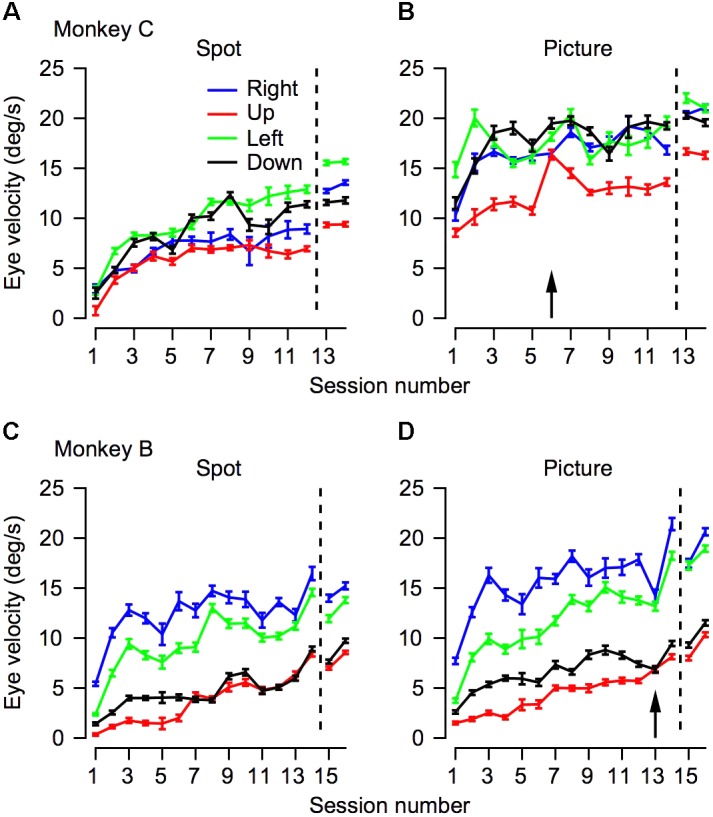
The progression over training days for eye velocity during pursuit initiation. The horizontal axis represents the session number. Values to the right of the vertical dotted line were recorded after extensive training on pursuit tasks. The vertical axis represents the average eye velocity at 250 ms after target motion onset. The top **(A,B)** and bottom **(C,D)** plots shows data for Monkeys C and B. Different columns correspond to the different target conditions and different colors represent different movement directions. The arrows in **(B,D)** mark the sessions in which we switched to the second monkey picture.

**FIGURE 5:**
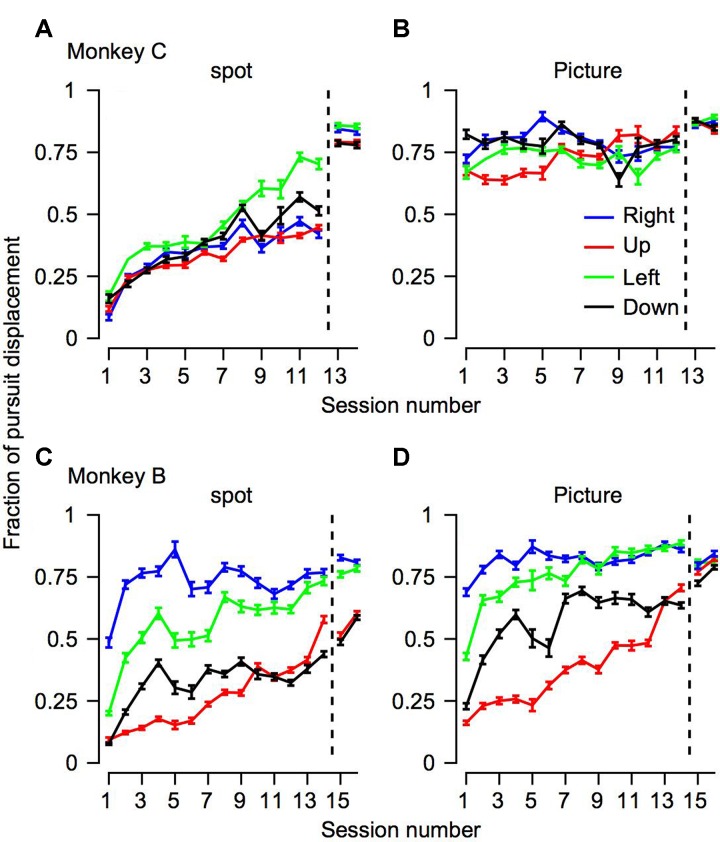
The progression over training days for the fraction of pursuit displacement across all the trials. The progression over training days of the fraction of position displacement with pursuit. The horizontal axis represents the session number, sessions to the right of the vertical dotted line were recorded after extensive training on pursuit tasks. The top **(A,B)** and bottom **(C,D)** plots shows data for Monkeys C and B. Different columns correspond to the different target conditions and colors represent the movement directions.

**FIGURE 6:**
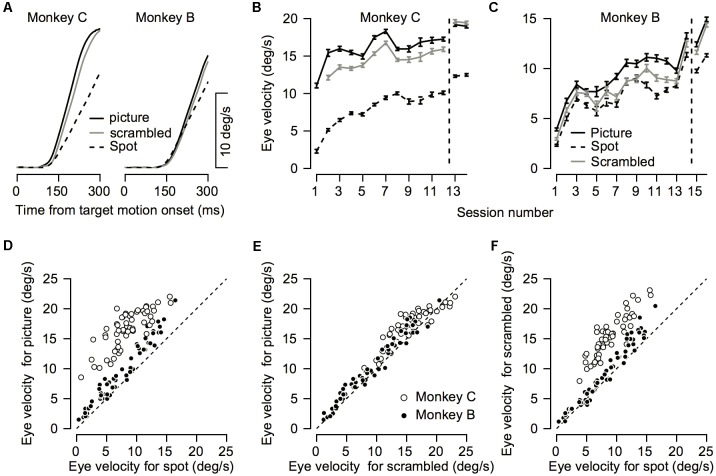
Comparison between pursuit initiation for the different targets. **(A)** Average eye velocity traces across all recording sessions for the picture (solid black), scrambled (gray), and spot (dashed) targets. Left and right plots show data for Monkeys C and B. **(B,C)** The progression over training days for eye velocity during pursuit initiation. The horizontal axis represents the session number. Values to the right of the vertical dotted line were recorded after extensive training on pursuit tasks. The vertical axis represents the average eye velocity across all target motion directions at 250 ms after target motion onset. **(D–F)** Quantitative comparison between target conditions. Each symbol shows the eye velocity at 250 ms after target motion onset; different symbols correspond to different sessions and different motion directions. Open and filled circles show the data for different monkeys. The plots present comparisons of the spot vs. the picture **(D)** the scrambled picture vs. the monkey picture **(E)**, and the spot vs. the scrambled picture **(F)**.

**FIGURE 7:**
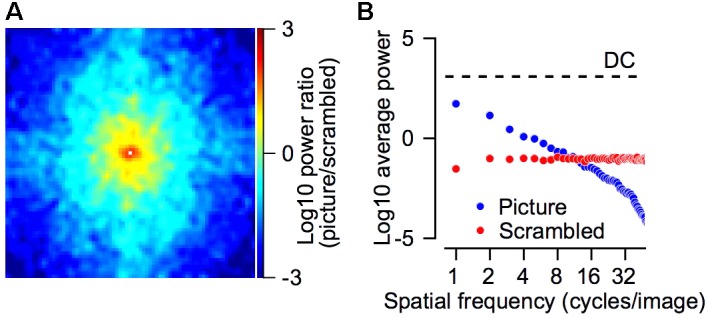
Power spectrum of the picture and scrambled stimuli. **(A)** The log_10_ of the ratio between the power spectrum of the picture and the scrambled target. Positive values (picture > scrambled) are represented by red/yellow pixels and negative values (picture < scrambled) in cyan/blue colors. The power spectrum was smoothed with a 2-dimensional Gaussian kernel (*SD* = 1 pixel). The DC component was removed from this plot and replaced by a small white square in the center of the plot. **(B)** The average power as a function of the spatial frequency for the picture (blue) and scrambled (red) stimuli. The dashed line regresses the DC component, which was equal for both stimuli. The spatial frequencies were calculated by transforming the power spectrum into polar coordinates (see section “Materials and Methods”). **(A,B)** Shows the power spectrum of the grayscale image.

**FIGURE 8:**
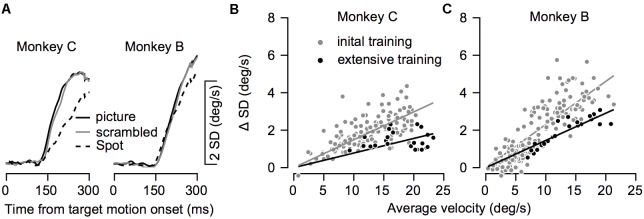
The variability in eye velocity during training. **(A)** The average standard deviation (SD) of the eye velocity across all recording sessions for the picture (solid black), scrambled (gray), and spot (dashed) targets. Left and right plots show data for Monkeys C and B. **(B,C)** The SD as a function of the mean eye velocity in all conditions before and after extensive training. Each dot shows data from a single target condition in a single direction of movement and on a single recording day. Gray and black dots show data before and after extensive training. Lines show the regression through the origin and correspond to the colors of the dots.

In the following results sections, we first analyze the differences in pursuit initiation (**Figures 2**–**4**) and then the full target motion epoch (**Figure 5**). In these sections we focus on the spot and picture stimuli. We then compare pursuit in all three stimulus conditions including the scrambled version of the picture (**Figures 6**, **7**). In the last section of the results, we show how training affects variability in these movements (**Figure 8**).

### The Initiation of Smooth Pursuit Eye Movements Improves Over Days of Training

The improvement across training was highly apparent in the smooth pursuit initiation. **Figure 2** depicts the average eye velocity at pursuit initiation when the stimulus was either a spot (gray) or a picture (black), for both monkeys. To calculate the eye velocity, we smoothed the eye position with a Gaussian kernel with a standard deviation of 5 ms and digitally differentiated the position signal. On the first training day the average eye velocity was the slowest for the spot target (**Figure 2**, dashed gray). For the picture target, the eye accelerated markedly and almost reached target velocity (**Figure 2**, dashed black), indicating that as early as the 1st day of pursuit, the monkeys could track the target in the step-ramp paradigm. The monkeys were trained on various pursuit paradigms for half a year, after which we reexamined pursuit behavior. After this extensive training period, pursuit initiation was enhanced markedly when tracking a moving spot (**Figure 2**, solid gray line) and improved for the picture target as well (**Figure 2**, solid black line). However, even after extensive training, acceleration was still lower for the spot target (**Figure 2**, solid black vs. solid gray). Note that the examples in **Figure 2** show one direction of movement; in later sections, we analyze eye movements in different directions separately. Thus, the examples indicate that the monkeys could track some stimuli with a large gain (speed/target velocity) as early as the 1st day of training and that even after extensive training there was still a difference in the pursuit of a picture and a spot target.

To test to what extent the monkeys improved their pursuit over training and whether the improvement depended on the nature of the visual stimulus, we measured eye velocity during the first 12–14 days of training on a step-ramp paradigm. Overall monkey B and C performed an average of 1365 (±552, SD) and 850 (±435, SD) trials per day. In both the picture and spot conditions the monkeys tended to have lower gains during the initial training days, as indicated by the average eye velocity depicted by the blue traces in **Figures 3A,B,E,F**. As training progressed the gain increased, as shown in **Figure 3** by the tendency of the green and red traces to lie above the blue traces. After the initial training, the monkeys were engaged in a variety of different tasks for several months (similar for the most part to the tasks described in Joshua and Lisberger, 2012). After this period, we resampled pursuit and found a further increase in pursuit gain, as indicated by the black dashed traces in **Figure 3** that tend to lie above all the other traces.

Both monkeys exhibited similar trends in terms of improvement across days; however, there were some differences between them. For Monkey C the gradual increase was the most pronounced for the spot condition (**Figure 3A**). In the picture condition, there was an increase in the pursuit velocity between the 1st and 2nd day of training, but in further training the increase tapered off (**Figure 3B**). Extensive training led to a small additional increase (black dashed traces in **Figures 3A,B**). For Monkey B there was a gradual increase in eye velocity in both stimulus conditions (**Figures 3E,F**). Monkey B showed larger gains for pursuit in the picture vs. spot condition; however, the difference was not as pronounced as for Monkey C.

To quantify the improvement across days we decomposed the average eye traces into a linear increase in eye velocity (constant acceleration) after initiation (**Figures 3C,G**, see section “Materials and Methods”) and the latency from motion onset (**Figures 3D,H**). The increase in the eye velocity traces across days of training (**Figures 3A,B,E,F**) resulted from an increase in eye acceleration (regression slope with session number > 0, *p* < 0.05) and a decrease in the latency of the movement (regression slope with session number < 0, *p* < 0.05). In both monkeys the acceleration was larger for the picture than for the spot target (**Figures 3C,G**, black vs. gray line, *p* < 0.01 Wilcoxon signed-rank test). The effect of the latency was inconsistent across the two monkeys. For Monkey B we found longer latencies for the picture vs. the spot (**Figure 3H**, *p* < 0.01 Wilcoxon signed-rank test) whereas for Monkey C there were no consistent differences (**Figure 3D**, *p* > 0.05). The latency values for Monkey B might seem inconsistent with the larger overall gain of the pursuit for picture vs. spot (**Figure 2**). Note, however, that the overall effect of constant acceleration on the eye velocity was much larger than the effect of latency, since acceleration but not latency accumulates in time.

### Anisotropies in Pursuit Are Maintained Across Training

Humans and monkeys have anisotropies in pursuit velocity (Grasse and Lisberger, 1992; Takeichi et al., 2003; Ke et al., 2013). Typically, after training monkeys tend to have a larger gain for horizontal pursuit and a lower gain for vertical pursuit, such that upward pursuit has the lowest gain (see for example Joshua and Lisberger, 2012). We measured eye velocity from the 1st day of training in the four cardinal directions to test whether and to what extent these anisotropies would develop across training. We used the eye velocity at 250 ms after motion onset for further analysis. Other quantifications of the eye velocity during initiation such as the average velocity in the first 250 ms after motion onset or velocity at 200 ms did not alter the findings. The results of this analysis are presented in **Figure 4**. The effects of training depicted in the average traces in **Figure 3** can be seen in **Figure 4** in the tendency of the traces to increase across sessions.

There were clear anisotropies in eye velocity from the 1st day of training (**Figure 4**, *p* < 0.05 in both monkeys and in all stimulus type conditions; one-way ANOVA). In both monkeys and in all conditions the pursuit velocity was the lowest for upward movement (red traces in **Figure 4**). With a few exceptions, across training, the anisotropies were consistent. This is indicated by the tendency of the different traces in **Figure 4** not to intersect. One exception to the overall consistency of the anisotropies was the crossover of the traces in conditions where target velocity was close to the eye velocity (i.e., when the eye velocity was close to 20°/s, **Figure 4B**). In these conditions, the ceiling effect of the target velocity probably masked the anisotropies. Another minor exception to the overall tendency of the anisotropies to be the same across training was that after extensive training the order of the gain changed in Monkey C (in **Figure 4A**, the blue and black traces reversed order to the left and right of the dashed line). This could be a result of the extensive training schedule. During the initial training, we equalized the number of trials for the different directions; hence, we can be confident that we did not confound the anisotropies with biases that might arise from unbalanced training. Later the anisotropies could have been affected to some extent by the training schedule where we did not attempt to equalize the direction of movement.

Analyzing the eye traces separately for each direction also revealed a potential effect of switching the picture stimulus. In monkey C we switched pictures at the start of the 6th day. Immediately after the switch there was an increase in eye velocity for upward movement (red line in **Figure 4B**). In monkey B the opposite trend was observed; i.e., on the day we switched pictures (day 13) there was a small decrease in eye velocity (**Figure 3D**). Interestingly for both monkeys eye velocity rebounded the day after the switch: it decreased for monkey C and increased for monkey B. Future work utilizing a series of pictures while probing pursuit could systematically explore the effects of picture content and novelty on pursuit.

### Interaction Between Pursuit and Saccades

Up to this point we have focused on the initiation of pursuit that corresponds to the open loop period. Because saccades have longer latency than pursuit in the period we have analyzed so far, there were few saccades; however, later in the trials the monkeys often made saccades to compensate for inapt pursuit gain (see for example **Figures 1C–F**). A previous study showed that on the 1st day of training the fraction of movement with pursuit corresponded to approximately 20% of the overall target displacement (Bourrelly et al., 2016). That study focused on tracking initiated with a saccade with small stimuli (0.4°). Here we expanded the stimulus set and in addition to a small spot (0.5°) also presented larger pictures. We found that picture stimuli can drive initiation with pursuit from the 1st day of training (**Figures 1–4**). Hence next we tested whether the stimuli we used could drive tracking with pursuit from Day 1, or as was found previously, that tracking is initially accomplished mostly through saccades.

To quantify how much of the tracking involved smooth pursuit, for each trial we calculated the fraction of displacement through pursuit vs. the total eye displacement (Bourrelly et al., 2016). Values close to zero in this analysis correspond to tracking that is mostly accomplished via saccades and values close to one correspond to tracking mainly by pursuit. On Day 1 of training the fraction of pursuit displacement was larger for the picture than the spot condition (left-most value in **Figures 5A** vs. 5B,5C vs. 5D, *p* < 0.01 two- way ANOVA). This difference between the picture and the spot on Day 1 was very large for Monkey C. Although smaller, there still was a substantial increase in pursuit displacement for Monkey B. For example, when the stimulus moved to the left (green traces in **Figure 5**), on the 1st day Monkey B tracked 20% of the displacement with pursuit in the spot condition and 42% for the picture condition. Across all motion directions, on the 1st day the average pursuit displacement for the spot was 21 and 10% for monkeys B and C, respectively. The values for the picture targets were 37 and 72% for Monkeys B and C. Thus, these results are consistent with previous reports that displacement of small spots are tracked initially primarily by saccades but also indicate that a lack of pursuit tracking is not the result of an inherent limitation of the pursuit system.

Overall, the findings thus indicate that the effects of training, stimulus condition and motion direction were similar for pursuit initiation (**Figure 4**) and the fraction of pursuit displacement (**Figure 5**). For each training day we calculated the average fraction of pursuit displacement separately for different target direction conditions and stimulus conditions. In both monkeys the fraction of pursuit displacement increased across days of training (**Figures 5A–D**). The improvement was substantial for both monkeys in the spot condition. In the picture condition, the fraction of pursuit displacement in Monkey C reached a plateau of around 75% as early as the 1st day of measurements (**Figure 5B**), whereas Monkey B required a few more days of training to achieve values close to 75% for horizontal movement and longer training was required for vertical movement (**Figure 5D**). Again, this is consistent with the lower gain observed for Monkey B in the vertical movement and the lower gain for Monkey B compared to C for the picture stimulus.

We found an effect similar to the one we found in pursuit initiation and the fraction of pursuit displacement for the amplitude of the first saccade. On the 1st day the average amplitude was larger for the spot than for the picture condition. In the spot condition on the 1st day the average amplitude of the first saccade was 4.1° (±2.2, SD) and 5.72° (±2.8) for Monkeys B and C. The values for the picture targets were smaller; 3.1°(±1.8) and 2.6° (±1.6) for Monkeys B and C. Training reduced the amplitude of the first saccade. After extensive training the average amplitude of the first saccade for the spot condition was 1.94° (±0.81) and 1.2° (±0.63) for Monkeys B and C. The values for the picture targets were smaller; 1.29° (±0.71) and 0.77° (±0.54) for Monkeys B and C.

### Intermediate Gain for a Stimulus With the Size and Luminance of the Monkey Picture

The differences in pursuit between the monkey picture and the spot could be attributed to either the difference in the relevance of the stimulus to the monkey prior to training, and/or the low level properties of the stimulus such as its size (Heinen and Watamaniuk, 1998) or its spatial frequency components (Adelson and Bergen, 1985; Simoncini et al., 2012; Gekas et al., 2017). We chose the monkey picture to optimize the pursuit conditions since the monkey picture was not only relevant to monkeys but also larger than the spots and contained motion signals at various frequencies. To test whether the larger gain of the pursuit was related solely to the object in the picture or might also be the result of the lower level properties of the stimulus such as its size or overall luminance, we compared the pursuit of the spot, picture and a scrambled version of the picture (**Figure 1B**). Note that the main aim of the current study was to optimize stimuli for enhancing tracking from the 1st day. Therefore, the number of conditions we could test was limited and we did not attempt to perform an extensive study to explore the properties of the monkey picture that drove faster pursuit.

We found that eye velocity was the fastest for the picture condition, slightly slower for the scrambled picture, and the slowest for the spot (**Figure 6A**). To quantify the effect, we calculated the eye velocity 250 ms after target motion onset. In both monkeys we found a significant difference between the stimulus conditions (repeated-measure ANOVA *p* < 0.01). *Post hoc* comparisons (Tukey’s *post hoc* comparison *p* < 0.01) revealed that the eye velocity was indeed the fastest for the picture condition, slightly slower for the scrambled picture, and the slowest for the spot (**Figures 6D–F**). This order of eye speeds was maintained across first 12–14 training days (**Figures 6B,C**). An exception to this order was that after extensive training, pursuit in monkey C for the scrambled picture was slightly greater than the velocity for the picture (right most data in **Figure 6B**). Note as well that we did not present the scrambled condition on the 1st day of training for Monkey C (see the missing gray data point in **Figure 6B**).

For both monkeys the gain increase in pursuit in the picture vs. the scrambled condition was similar. The average across all sessions for the velocity ratio between the scrambled and picture condition (scrambled/picture) was 88 and 90% for Monkeys B and C. The velocity ratio between the spot and the picture condition was smaller for Monkey C (spot/picture eye velocity = 50%) than Monkey B (spot/picture = 76%). Thus, the faster pursuit for scrambled vs. spot indicates that the pursuit enhancement we found in the picture condition was not only related to the object in the picture. The faster pursuit for the picture vs. the scrambled stimulus suggests that the object (monkey picture) potentiated the pursuit, but other explanations are possible. Nevertheless, one crucial difference between the picture and scrambled stimuli has to do with their spectral frequency components.

The picture had greater power at low frequencies whereas the scrambled target had greater power in the higher frequency range. **Figure 7** compares the power spectrum of the picture and scrambled targets. We calculated the power spectrum (see section “Materials and Methods”) of the picture and the scrambled version that are shown in **Figure 1B**. For each frequency in the 2-dimensional spectrum, we calculated the ratio between the power of the picture and scrambled targets. The larger power spectrum of the picture at low frequencies is shown by the tendency of the pixels to plot in yellow and red around the center of **Figure 7A**. The smaller power spectrum of the picture in the higher frequencies appears in the tendency of the pixels close to the margins of **Figure 7A** to plot in cyan and blue. We transformed the power spectrum into polar coordinates (see section “Materials and Methods”) and averaged the power across all spatial frequencies (**Figure 7B**). In the frequency range between 1 and 8 cycles per image, the power of the picture was greater whereas in frequencies above 8 cycles per image the power of the scrambled stimuli was greater. We return to these points in the discussion.

### Patterns of Variability During Initial Training and After Extensive Training

So far, we have shown how training affects average eye velocity. Below we focus on the variability of movement. We calculated the standard deviation of the eye velocity during movement. The patterns of progression of the standard deviation in time were similar to the average eye velocity patterns (**Figures 6A**, **8A**). The standard deviation increased when the eye started to move and was larger for pictures and scrambled targets than for the spot. This pattern is consistent with the signal-dependent noise that is often observed in motor control (Harris and Wolpert, 1998).

To test whether training affected the variability beyond the effect it had on average eye velocity we compared the average and standard deviation of the eye velocity at 250 ms after motion onset. To focus on the pursuit component, we subtracted the standard deviation prior to the movement from the standard deviation during movement. **Figures 8B,C** show the standard deviation as a function of the average eye velocity, where each data points represents a single experimental condition (across days, movement direction and stimulus type). The values after extensive training tended to lie below the values during training, indicating that conditions with similar average velocity tended to have lower standard deviations after extensive training. The regression through the origin (0,0) had a smaller slope after extensive training (gray and black lines in **Figures 8B,C**). The slope estimates the coefficient of variation for the eye velocity before and after extensive training (0.15 and 0.08 for Monkey C, 0.23 and 0.14 for Monkey B). Thus, training increased the precision of eye velocity since there was less variability per average eye velocity after extensive training. Note that because of the substantial noise in the analysis of the coefficient of variation, especially when the signal is very small, we focused on comparison before and after extensive training and not on the modulation of the coefficient of variation during the initial training.

## Discussion

We analyzed the eye movements of monkeys from the 1st day of training on a step-ramp task to assess the conditions that enabled them to exhibit pursuit from the initial days of training. The results show that under the appropriate conditions, the monkeys had a high pursuit gain in the step-ramp paradigm as early as the initial days of training. When the monkeys tracked a picture, the pursuit gain was high, and training rapidly enhanced the gain. Tracking of small spots was associated with lower gain, but increased substantially over the 12–14 days of training. We conducted control experiments which showed that the difference between the picture and the spot was influenced by the low-level properties of the stimulus such as its size, but some of the effect could have been due to the object in the picture.

There are crucial differences between smooth pursuit in the laboratory and in the natural environment. In natural environments gaze shifts are obtained by coordinating head and eye movements (Bizzi et al., 1971; Roy and Cullen, 1998), whereas in lab experiments, behavior is often restricted to eye movements alone. This difference in the monkey’s mobility also strongly influences sensory inputs. Fixating the head precludes motion flow (Warren and Hannon, 1988) and most vestibular inputs (Roy and Cullen, 1998). The properties of the visual stimuli we presented here were also different from natural conditions. For example, in natural conditions the segregation between target and background is usually not as sharp as in our experiments. Thus, our findings indicate that these potentially major differences between the natural and laboratory environments do not place a strong constraint on monkeys’ pursuit ability and that laboratory pursuit indeed approximates the natural repertoire of the monkey.

Previous work has indicated that training markedly enhances smooth pursuit eye movements (Bourrelly et al., 2016) and ocular following responses (Hietanen et al., 2017). Bourrelly et al. (2016) found that on the initial day of training the percent displacement of the eye attributed to pursuit was very small (20% of target displacement). We extended this work by showing that this result depends on the type of visual stimulus. Hence, when the gain is low, it appears to be the result of the specificities of the visual stimuli as opposed to other laboratory setup factors such as restraining the head or the posture of the monkey. The consistency between experiments for the spot stimulus indicates that differences between experiments may not be crucial factors. Specifically, the large displacement step (16°) implemented by Bourrelly et al. (2016) should not be interpreted as the cause of the low gain of pursuit. We used the step-ramp paradigm with step eccentricities that are known to drive pursuit effectively (Lisberger and Westbrook, 1985). Therefore, even in conditions that are favorable for initiating pursuit movement, at the start of training the monkey mostly tracked spots with saccades.

The consistency between the current and previous experiments further validates the approach we and others have taken. One potential confounding factor affecting previous experiments on monkeys has to do with the mechanical interference of the eye coil, which could affect eye movements and lead to anisotropies. In the current study, we used camera-based eye tracking. The consistency of the current findings with other studies with respect to movement anisotropies (Grasse and Lisberger, 1992; Joshua and Lisberger, 2012) indicates that mechanical interference was not the source of anisotropies, and validates the approach used in previous experiments.

### Pursuit of Objects and Patterns

We used pictures of monkeys to engage the natural tendencies of the monkeys to direct their gaze toward conspecific stimuli. The pursuit system does not depend solely on low level sensory features. For example, the size of the reward can determine the gain of the pursuit (Joshua and Lisberger, 2012; Brielmann and Spering, 2015; Schutz et al., 2015). Therefore, we expected that objects that were significant to the monkeys before training would potentiate pursuit. The slower pursuit in the scrambled condition suggests that the object itself was important for doing so. Nevertheless, other interpretations are possible. The pictures contained low level features that are favorable to pursuit. The large size and non-uniformity of the picture in color, the contrast, and the texture all insured that there were strong motion signals when the picture moved. The slower pursuit in the scrambled condition might have resulted from features that distinguished the picture from the scrambled picture which might also have influenced the pursuit. The scrambled picture was equal to the picture in term of size, overall luminance and color but contained stronger high frequency and weaker low frequency spatial components (**Figure 7**). Since the goal of this study was to study whether and to what extent monkeys learn to pursue in a laboratory setup, our ability to characterize the features of the pictures that led to the high gain was limited. These could be low level features such as the overall energy in different frequency channels (Simoncini et al., 2012) or the textures of the picture (Portilla and Simoncelli, 2000) or more abstract high level features such as the existence of a face in the picture.

Although the current study does not unequivocally show that the presence of the monkey image rather than the pictures’ low-level parameters potentiate pursuit, it does point to a way to test this notion. The difference between pursuit of a face and a scrambled picture, together with the algorithms that can be used to extract specific features of the picture (Portilla and Simoncelli, 2000; Yoonessi and Kingdom, 2008) could be used to determine whether the enhancement in pursuit was due to the object in the picture or not. For example, comparing pursuit of pictures and a picture that maintains the texture but removes the face of the monkey could provide indications as to the importance of the object in the pursuit gain for monkeys. Comparing the monkey picture with other natural objects could indicate the specificity of the pursuit enhancements.

Another possibility is that the enhancement in the pursuit initiation was related to the subjects’ state at the time of pre-motion fixation. In the current experiment the object appeared before the target motion (**Figure 1A**). The enhancement in pursuit initiation could partly have been due to the preparatory mechanism (Mahaffy and Krauzlis, 2011; Raghavan and Joshua, 2017). These could reflect differences in the motivational state of the animal that might focus attention on the picture but less on the spot or scrambled stimuli. The effect of the pre-motion attention could be tested by pairing different targets for fixation and motion. Tasks that probe attention during tracking (e.g., Lovejoy et al., 2009) might help disentangle the role of focused attention during steady state pursuit.

## Conclusion

We found that monkeys can perform a step-ramp task relatively well from the initial days of training. Even when the initial gain was small, the monkeys adjusted rapidly over the 12–14 days of training. This indicates that this paradigm does not require highly specialized mechanisms that might be generated by extensive training. Thus, it is likely that the results obtained from the step-ramp paradigm generalize across animals and from monkey to humans.

## Author Contributions

YB carried out the experiments with support from MY. MJ analyzed the data with support from YB. MJ wrote the manuscript with support from YB and MY. MJ conceived the original idea and supervised the project.

## Conflict of Interest Statement

The authors declare that the research was conducted in the absence of any commercial or financial relationships that could be construed as a potential conflict of interest.
